# Antiviral Activity of Porcine Interferon Regulatory Factor 1 against Swine Viruses in Cell Culture

**DOI:** 10.3390/v7112913

**Published:** 2015-11-17

**Authors:** Yongtao Li, Hongtao Chang, Xia Yang, Yongxiang Zhao, Lu Chen, Xinwei Wang, Hongying Liu, Chuanqing Wang, Jun Zhao

**Affiliations:** College of Animal Husbandry & Veterinary Science, Henan Agricultural University, Zhengzhou 450002, China; yongtaole@126.com (Y.L.); ndcht@163.com (H.C.); liyihan2001@163.com (X.Y.); zyx0920@163.com (Y.Z.); chenluhau@sina.com (L.C.); xinweicliff@126.com (X.W.); ndlhy@163.com (H.L.); wchuanq@163.com (C.W.)

**Keywords:** porcine IRF1, antiviral activity, IFN-β, IFN-stimulated genes

## Abstract

Interferon regulatory factor 1 (IRF1), as an important transcription factor, is abundantly induced upon virus infections and participates in host antiviral immune responses. However, the roles of porcine IRF1 (poIRF1) in host antiviral defense remain poorly understood. In this study, we determined that poIRF1 was upregulated upon infection with viruses and distributed in nucleus in porcine PK-15 cells. Subsequently, we tested the antiviral activities of poIRF1 against several swine viruses in cells. Overexpression of poIRF1 can efficiently suppress the replication of viruses, and knockdown of poIRF1 promotes moderately viral replication. Interestingly, overexpression of poIRF1 enhances dsRNA-induced IFN-β and IFN-stimulated response element (ISRE) promoter activation, whereas knockdown of poIRF1 cannot significantly affect the activation of IFN-β promoter induced by RNA viruses. This study suggests that poIRF1 plays a significant role in cellular antiviral response against swine viruses, but might be dispensable for IFN-β induction triggered by RNA viruses in PK-15 cells. Given these results, poIRF1 plays potential roles in cellular antiviral responses against swine viruses.

## 1. Introduction

Virus infection and subsequent replication poses a significant threat to the host immune system. As a result, every living species has evolved mechanisms for recognizing viruses and counteracting them through immune responses. Type I interferons (IFN) are the central components of early innate immune response against viral infection. Generally, when a virus is invading a cell, cellular pattern recognition receptors (PRRs) can recognize viral nucleic acids, and transmit signals to downstream adaptors, which eventually results in the activation of transcription factors of IFN regulatory factor (IRF) family, primarily including IRF3 and/or IRF7, to induce the expression of type I IFN [1]. Type I IFN bind a heterodimeric transmembrane receptor termed the IFNα receptor (IFNAR) to activate interferon-stimulated gene factor 3 (ISGF3) via the JAK-STAT signaling pathway and induce the coordinated upregulation of hundreds of interferon-stimulated genes (ISGs) that orchestrate an antiviral state in the cells [2].

In addition to IRF3/7, IRF1 also plays a significant role in type I IFN induction in cell-specific fashions. As the first member of IRF family, IRF1 is constitutively expressed in most cell types and is dramatically induced upon viral infection, treatment with dsRNA or IFN stimulation [3,4,5]. Early research has indicated that IRF1, as an important mediator, regulates the expression of type I IFN [4,6,7]. Subsequently, analysis of IRF1^−/−^ cells from mice has shed light on the unnecessary role of IRF1 in IFN-β induction by virus infection [8,9,10]. Although, whether IRF1 is directly involved in IFN induction remains controversial, increasing studies show that IRF1 plays a pivotal role in host antiviral responses against a wide set of viruses [11,12,13,14,15]. Recently, systematic screening of antiviral effectors has identified human IRF1 as a broadly acting effector to defend against diverse viruses tested, highlighting the physical relevance of IRF1 in the innate antiviral responses [16,17].

In our previous studies, transcriptome profiling analyses showed that ISGs were the most highly induced genes in pig lungs after H1N1 influenza virus infection at an early stage [18]. We investigated the antiviral activity of porcine ISGs and initially identified porcine IRF1 (poIRF1) as the most active effector in the defense against influenza virus. Although many studies have already focused on antiviral functions of human or mouse IRF1, the antiviral functions of poIRF1 are poorly understood. Therefore, we sought to investigate the antiviral activity of poIRF1 against four swine viruses in cultured porcine kidney epithelial cells (PK-15) and investigated the possible mechanism by which IRF1 exerts its antiviral effects. This study suggests that poIRF1 plays a significant role in cellular antiviral response against swine viruses, but might be dispensable for IFN-β induction triggered by RNA viruses in PK-15 cells.

## 2. Results

**Figure 1:**
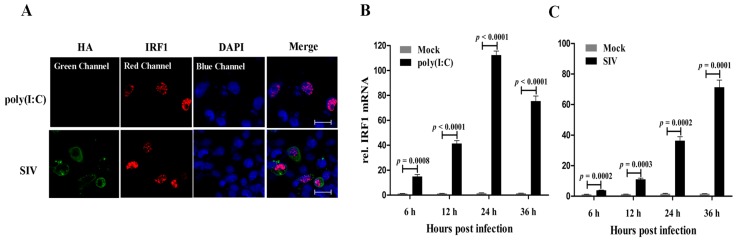

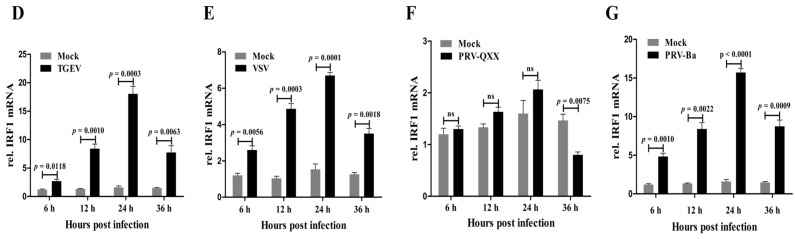
poIRF1 is induced in PK-15 cells upon dsRNA stimulation and viral infections. (**A**) Detection of poIRF1 and influenza virus Hemagglutinin (HA) protein by immunofluorescence microscopy. PK-15 cells on coverslips were treated by poly(I:C) stimulation or SIV infection. At 12 h post-treatment, IFA was performed for visualization of the distribution of poIRF1 and HA protein detected by Cy3- and FITC-labeled secondary antibodies respectively and visualization of cell nuclei stained by DAPI. Confocal images were captured on a LSM510 META microscope; scale bar, 50 µm; (**B**–**G**) The expression patterns of poIRF1 in PK-15 cells stimulated with poly(I:C) or infected with viruses; PK-15 cells were stimulated with poly(I:C) (**B**) or infected with SIV (**C**); TGEV (**D**); VSV (**E**) and PRV-QXX (**F**) and PRV-Ba (**G**). At the indicated times, the quantification of poIRF1 transcripts was performed by RT-qPCR at 6, 12, 24 and 36 hpi. Relative quantitative values of poIRF1 gene were normalized to the level of GAPDH. Differences of gene expression between infected and control cells were analyzed by using the Student’s *t*-test. The results were plotted in graph format as mean ± SD.

Firstly, the subcellular localization of poIRF1 protein was investigated in PK-15 cells upon poly(I:C) or SIV infection. As shown in Figure 1A, poIRF1 (in red) accumulated primarily in nucleus of both poly(I:C)- and SIV-stimulated cells. To determine the expression patterns of poIRF1, PK-15 cells were stimulated with poly(I:C) or infected with swine influenza virus (SIV), vesicular stomatitis virus (VSV), pseudorabies virus (PRV) and transmissible gastroenteritis virus (TGEV), and poIRF1 transcripts were measured by RT-qPCR at various time points. Our data showed that IRF1 mRNA was expressed at a low basal level in PK-15 cells, and was induced rapidly after poly(I:C) treatment at very considerable levels at 6 hours post infection (hpi) and reached to the peak at 24 hpi (72-fold *versus* control) (Figure 1B). In SIV-infected cells, poIRF1 increased continuously and remained high at 36 hpi (45-fold *vs.* control) (Figure 1C).

In TGEV-infected cells, poIRF1 increased continuously and reached to the peak at 24 hpi (11-fold *versus* control) (Figure 1D). However, poIRF1 exhibited only a slight increase in response to VSV infection, with the highest expression levels (4.5-fold *vs.* control) at 24 hpi (Figure 1E). In addition, poIRF1 expression level exhibited large differences in the virulent PRV-QXX (not significant change) and attenuated PRV-Ba (10-fold *vs.* control at 24 hpi) infected cells (Figure 1F,G), indicating the possible relationship between viral virulence and the antagonistic capability of virus to IRF1 expression. In brief, the expression patterns of poIRF1 induced by different viruses in PK-15 cells suggested that poIRF1 might exert different antiviral activities against these swine viruses.

**Figure 2:**
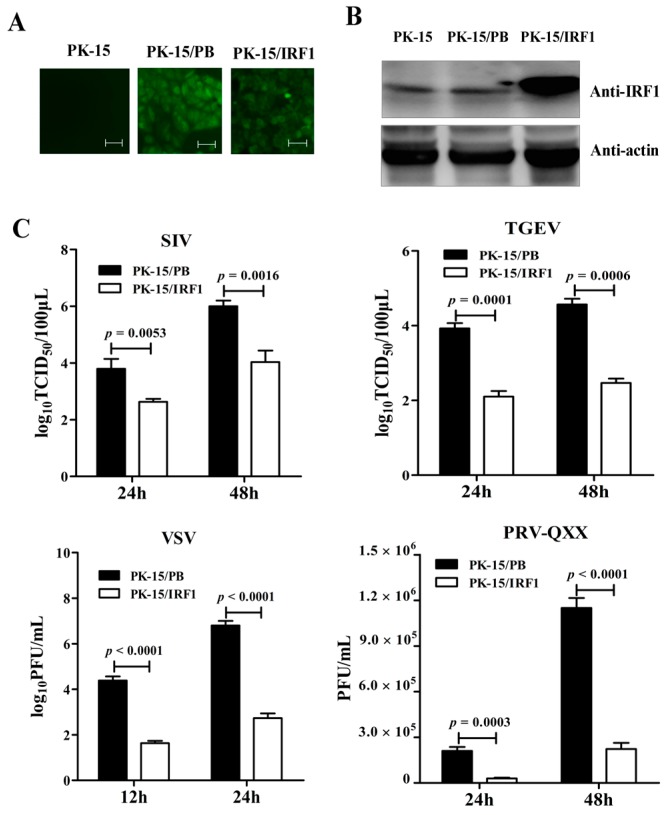
Overexpression of poIRF1 potently inhibits virus replication. (**A**) PK-15 cells stably expressing poIRF1 were established by PB transposon systems. After puromycin treatment, the single puromycin-resistant and GFP-positive cell clones, designated as PK-15/IRF1 and PK-15/PB, were captured on a fluorescence microscope; scale bar, 100 µm; (**B**) Detection of poIRF1 protein by Western blot. Protein lysates from PK-15, PK-15/PB and PK-15/IRF1 were denatured in SDS sample buffer. Denatured lysates were analyzed by SDS-PAGE followed by Western blot using antibodies detecting poIRF1, as indicated. β-actin was used as a loading control; (**C**) The impact of poIRF1 overexpression on viral replication. PK-15 cells stably expressing poIRF1 or with vector alone were incubated with indicated viruses for 24 h and 48 h before supernatants were harvested. The viral titers in supernatants were measured with TCID_50_ assay or plaque assay.

In order to determine the effect of poIRF1 overexpression on virus replication, PK-15 cells stably expressing poIRF1 or mock cells were established by PB transposon systems, designed as PK-15/IRF1 and PK-15/PB respectively (Figure 2A). Western blot results indicated high level expression of poIRF1 in PK-15/IRF1 cells relative to PK-15/PB and normal PK-15 cells (Figure 2B). Then, PK-15/PB and PK-15/IRF1 cells were infected with swine viruses at a multiplicity of infection (MOI) of 0.1 TCID_50_/mL for SIV and TGEV, at a MOI of 0.1 PFU/mL for VSV and PRV-QXX. After infections, viral titers in the culture supernatants were determined at the indicated time points. The results showed that the SIV titers from PK-15/IRF1 cells were reduced by 14- and 100-fold compared with these from PK-15/PB cells at 24 and 48 hpi, respectively. TGEV titers from poIRF1-overexpressed cells were reduced by approximately 63-fold and 126-fold compared with those in PK-15/PB cells at 24 and 48 hpi, respectively. Moreover, detection of VSV-infected cells under microscope revealed readily detectable cytopathic effects (CPE) at 12 and 24 hpi in PK-15/PB, but rarely detectable CPE in PK-15/IRF1 cells (Figure not shown). In accordance with microscope detection, VSV titers in the cell supernatants revealed up to 630- and 10,000-fold reduction in PK-15/IRF1 cells compared with these in PK-15/PB cells at 12 and 24 hpi. We also tested poIRF1-mediated inhibition of a dsDNA virus, PRV, and detected obviously reduced CPE in PK-15/IRF1 cells compared with PK-15/PB cells. Plaque counts revealed markedly a 7- and 5-fold reduction of PRV titers in PK-15/IRF1 cells compared with PK-15/PB cells (Figure 2C). The results indicated that overexpression of poIRF1 potently inhibits virus replication.

**Figure 3:**
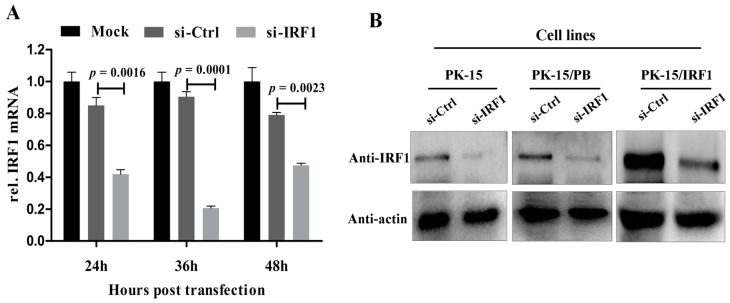

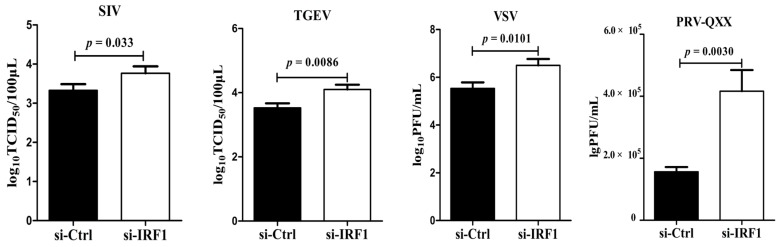
Knockdown of poIRF1 promotes moderately virus replication. (**A**) The knockdown efficiency of poIRF1 was determined by RT-qPCR. PK-15 cells were transfected with si-IRF1 or si-Ctrl for 24 h, 36 h and 48 h, and subsequently the poIRF1 expression in transfected cells was verified at mRNA levels by RT-qPCR; (**B**) The knockdown efficiency of poIRF1 was checked by Western blot. PK-15, PK-15/PB and PK-15/IRF1 cells were transfected with si-IRF1 or si-Ctrl for 36 h. The poIRF1 expression in transfected cells was verified at protein levels by Western blot assay. β-actin was used as a loading control; (**C**) The effect of knockdown of poIRF1 on viral replication. PK-15 cells were transfected with si-IRF1 or si-Ctrl for 36 h. Then, cells were incubated with indicated viruses for 24 h before supernatants were harvested. The viral titers in supernatants were measured with TCID_50_ assay or plaque assay.

To determine whether endogenous poIRF1 expression affects viral replication, siRNA targeting to poIRF1 (si-IRF1) was transfected into PK-15 cells with a non-targeting siRNA as control (si-Ctrl). The knockdown efficiency of poIRF1 was determined by RT-qPCR. The results showed that si-IRF1 transfection led to a 50%, 77% and 38% decrease in IRF1 transcription at 24, 36 and 48 h post transfection, respectively, compared with si-Ctrl (Figure 3A). As the IRF1 protein has a short half-life, IRF1 mRNA levels most probably correlate with IRF1 protein abundance [19]. Therefore, we further detected the knockdown effect of poIRF1 protein levels at 36 h post transfection and found that si-IRF1 transfection resulted to a significant decrease of poIRF1 protein in PK-15, PK-15/PB and PK-15/IRF1 cells compared with si-Ctrl (Figure 3B). Then, PK-15 cells were transfected by si-IRF1 and si-Ctrl for 36 h, and then infected with above swine viruses for determining titers in the culture supernatants at 24 hpi. The results showed that knockdown of poIRF1 approximately led to a 2.8-, 4.0-, 10- and 2.7-fold increase in viral growth for SIV, TGEV, VSV and PRV-QXX, respectively (Figure 3C). Taken together, our data suggest that knockdown of IRF1 enhanced moderately replication of SIV, TGEV, VSV and PRV in PK-15 cells.

**Figure 4:**
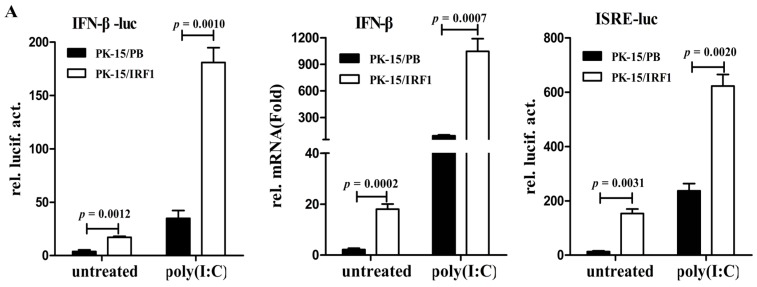

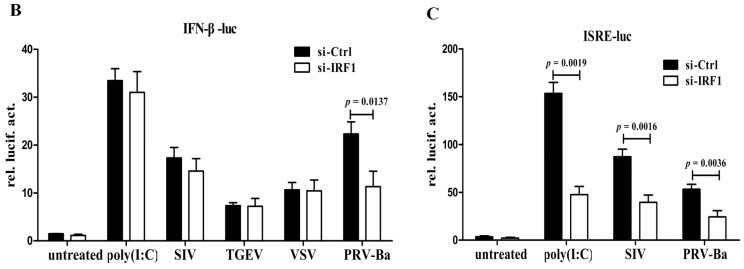
poIRF1 might be dispensable for IFN-β induction triggered by RNA viruses but essential for the activation of ISRE. (**A**) The effect of poIRF1 overexpression on activation of IFN-β and ISRE promoter. PK-15/PB and PK-15/IRF1 cells were cotransfected with indicated promoter constructs (0.5 μg) and pRL-TK (0.025 μg). At 24 h post-transfection, cells were transfected with poly(I:C) for another 24 h or left untreated and then harvested for detection of IFN-β and ISRE promoter activity. For measuring poIRF1-mediated induction of IFN-β, RT-qPCR was used to detect the transcription of endogenous IFN-β.The relative expression was normalized to the expression of GAPDH and expressed as fold induction relative to the expression level in control cells. Error bars represent SDs, which were obtained by measuring each sample in triplicate; (**B**,**C**) The effect of poIRF1 knockdown on activation of IFN-β and ISRE promoter. PK-15 cells were transfected with si-IRF1 or si-Ctrl for 36 h. Then, cells were cotransfected with IFN-β-luc or ISRE-luc and pRL-TK. At 24 h post-transfection, cells were transfected with poly(I:C) or infected with indicated viruses for another 12 h or left untreated and then harvested for detection promoter activity of IFN-β (**B**) or ISRE (**C**).

The induction of IFN response in virus-infected cells is an event central to innate antiviral immunity. To determine whether poIRF1 could regulate IFN-β response in PK-15 cells, we detected the contribution of poIRF1 to the induction of IFN-β and ISGs. As shown in Figure 4A, poIRF1 overexpression can result in significant activation of IFN-β promoter (4.5-fold) and upregulated expression of IFN-β transcript (8.4-fold) relative to that in control cells. Upon stimulation with poly(I:C), cells overexpressing poIRF1 produced significantly higher levels of IFN-β promoter activity and IFN-β mRNA than control cells. Meanwhile, we found that poIRF1 could also significantly enhance ISRE activation relative to that in control cells, and when followed by transfection of poly(I:C), ISRE activation was further promoted to a higher level (Figure 4A). On the contrary, poIRF1 knockdown did not alter IFN-β induction after poly(I:C) treatment or SIV, TGEV and VSV viruses infections, but significantly reduces PRV-induced IFN-β production (Figure 4B). Besides, the ISRE activation after poly(I:C) treatment or virus infections were also significantly reduced in poIRF1-knockdown cells (Figure 4C). These data suggest that poIRF1 might be dispensable for IFN-β induction triggered by RNA viruses but be essential for the activation of ISRE.

## 3. Discussion

It is well known that innate immune responses are critical for protecting host cells from early establishment of virus infection. IRF1 is an important mediator that regulates antiviral innate immune response. Although many studies have already focused on antiviral functions of human, mouse or fish IRF1 [20], the role of poIRF1 in controlling porcine viral infections is poorly understood. In this study, we showed that poIRF1 could potently inhibit the replication of four swine viruses in PK-15 cells and offered an insight into the possible mechanism by which IRF1 exerts its antiviral effects.

Many studies have showed the broad-spectrum antiviral activity of IRF1 in culture cells under overexpression or knockdown conditions. According to the literature report, overexpression of IRF1 can interfere with replication of a wide set of viruses, such as Newcastle disease virus (*Paramyxoviridae*) [21], vesicular stomatitis virus (*Rhabdoviridae*) [15,22], encephalomyocarditis virus (*Picornaviridae*) [23,24] and hepatitis C virus (*Flaviviridae*) [12]. Knockdown of IRF1 can enhance the replication of vaccinia virus (*Poxviridae*) [13], murine gammaherpesvirus 68 (*Gammaherpesviridae*) [14], vesicular stomatitis virus (*Rhabdoviridae*) [22] and West Nile virus (*Flaviviridae*) [23] by using IRF1-deficient mice or cells, but have no effect on replication of Newcastle disease virus [21]. In this study, we added a new conclusion that poIRF1 could also induce an antiviral state to three other types of virus, including SIV (*Orthomyxoviridae*), TGEV (*Coronaviridae*), and PRV (*Alphaherpesviridae*). It is worth noting that overexpression of poIRF1 can potently suppress the replication of viruses at different levels, whereas knockdown of poIRF1 can only promote viral replication moderately or slightly. Viral replication is strictly controlled by swine IFN-β in PK-15 cells [24,25,26]. Given the regulatory role of IRF1 in the transcription of IFN-β as well as ISGs *in vitro*, we therefore sought to investigate the impact of poIRF1 on IFN response.

As one of the ISGs, IRF1 is not a direct-acting antiviral effector but a typical modular against virus infections. To explore the possible mechanism by which poIRF1 limits the viral replication, we detected the contribution of poIRF1 to the activation of IFN-β and ISRE promoter. The results in reporter assays showed that overexpression of poIRF1 alone can stimulate the basal activity of the IFN-β promoter, and enhanced the activation of the promoter activity and transcriptional induction of the endogenous IFN-β in response to dsRNA. Our study also revealed that the absence of IRF1 did not clearly affect the induction of IFN-β in PK-15 cells in response to RNA virus infections but impaired the activation of ISRE, indicating that the antiviral phenotype of poIRF1 was not a consequence of IFN-β production and may have arisen directly through ISGs expression.

Recently, microarray analysis of IRF1-transduced cells lacking IFN signaling has showed that numerous ISGs were induced by IRF1, supporting the observation that IRF1 can activate IFN-independent pathways to induce antiviral states [17]. Several ISGs have been proposed as the mediators of the antiviral action of IRF1 to restrict replication of diverse virus families, such as Viperin and CH25H [11,14]. However, it is not clear exactly what specific effectors participate in the antiviral process mediated by IRF1. Comparative transcriptome analysis of virus infected wild type and IRF1^−/−^ cells is planned to identify these antiviral effectors preferentially induced by IRF1 that inhibit viral infections in a virus-type specific manner. Unraveling the antiviral mechanism of these effectors is therefore an important prerequisite to a better understanding of antiviral functions of IRF1.

On the other hand, increasing evidence has been presented that constitutive production of IFN-β could be detected in several cells in the absence of viral infection, albeit at a very low level [27,28]. AP-1 and NF-κB components are essential for constitutive IFN-β production [29,30,31,32]. Whether poIRF1 is involved in regulation of constitutive expression of IFN-β mediated by AP-1 and NF-κB components remains unclear. Moreover, as the immediate-early type of IFN, IFN-β is induced through the activation of constitutively expressed IRF3 protein in the early phase [33]. Once IFN-β is produced, it binds to its receptor, activates the JAK-STAT signaling pathway, and induces the expression of IRF7 [34]. In the late phase, IRF3 and IRF7 cooperate to amplify the induction of further and wider boost of Type I IFN responses [35]. The different roles of IRF1 in the early and late phases of Type I IFN responses in response to viral infections also require further explorations.

In conclusion, our study demonstrated that poIRF1 as an interferon-induced protein plays a significant role in cellular antiviral responses against four kinds of swine viruses, but might be dispensable for IFN-β induction triggered by RNA viruses in PK-15 cells. Given these results, poIRF1 plays potential roles in cellular antiviral responses against swine viruses.

## 4. Materials and Methods

### 4.1. Plasmids and Reagents

poIRF1(NM_001097413.1) was cloned into pMD-18T vector (TaKaRa, Dalian, China), and then subcloned into PiggyBac (PB) donor vector (System Biosciences, Mountain View, CA, USA). Porcine promoter reporter plasmids, IFN-β-luc and ISRE-luc were constructed according to previous study [36]. Mouse antiserum against poIRF1 and H1N1-HA were prepared previously in our laboratory. Monoclonal antibody against β-actin (Cali-bio, Coachella, CA, USA) was used for Western blot. The double-stranded RNA (dsRNA) analog poly(I:C) (Sigma, St Louis, MO, USA) was used at a final concentration of 2 μg/mL. Cells were transfected with indicated constructs in the presence of Lipofectamine 2000 according to the manufacturer’s instructions (Invitrogen, Carlsbad, CA, USA). The target sequences for poIRF1 knockdown and the negative control sequences are shown in Table 1. Small interfering RNAs (siRNA) corresponding to the target sequences were synthesized by a commercial company (Genepharma, Shanghai, China).

**Table 1:** Primers or sequences used in this study.

Name	Primer	Sequence (5′→3′)	Usage
poIRF1	PB-IRF1-F	ACTGAATTCATGCCCATCACTCGGATGCGCAT	PiggyBac donor vector construction
	PB-IRF1-R	TACCTCGAGGGCCTACGGTGCACAAGGAAT	
	IRF1-Q-F	GCACCAGCGACCTGTACAACT	RT-qPCR
	IRF1-Q-R	TCCTCATCTGTTGCAGCTTCA	
GAPDH	GAPDH-Q-F	TGCCAACGTGTCGGTTGT	RT-qPCR
	GAPDH-Q-R	TGTCATCATATTTGGCAGGTTT	
siRNA	si-IRF1	GGGCUGAUCUGGAUUAAUAdTdT	Knockdown
	si-Ctrl	UUCUCCGAACGUGUCACGUdTdT	

### 4.2. Cells and Viruses

PK-15 (Porcine kidney), VERO (African green monkey kidney), and MDCK (Madin-Darby canine kidney) cells were cultured with Dulbecco’s Modified Eagle Medium (DMEM) containing 10% FBS and incubated at 37 °C in 5% CO_2_. The SIV, VSV, virulent isolate QXX of PRV (PRV-QXX) and TGEV used in this study were isolated from pigs and stored in our laboratory. The attenuated PRV vaccine strain BarthaK61 (PRV-Ba) was obtained from a commercial company (Boehringer-Ingelheim, Ingelheim, Germany). All of these viruses can be grown in PK-15 cells.

### 4.3. Real-Time Quantitative PCR (RT-qPCR)

Cellular RNA extraction was performed using TRIzol by following the standard instructions (Invitrogen, Carlsbad, CA, USA). A volume of 2 µg of RNA was treated with DNase at 37 °C for 30 min to remove potential DNA contamination according to the manufacturer's instructions (Promega, Madison, WI, USA). Reverse transcription was performed using oligo(dT) primers. The specific primers of target genes were designed by Primer 5.0 (Table 1). The quantification of gene transcripts was performed by quantitative PCR using SYBR Green I dye (TOYOBO, Kyoto, Japan). Relative quantitative values of each gene were normalized to the level of Glyceraldehyde 3-phosphate dehydrogenase (GAPDH). Changes in gene expression were calculated by the ΔΔ*C*t method. Error bars represent standard deviations (SDs) from three separate experiments with assays performed in triplicate. Differences of gene expression between infected and control cells were analyzed using the Student’s *t*-test. The results were plotted in graph format as mean ± SD.

### 4.4. Western Blot

Cells were washed twice with PBS, lysed with cell lysis buffer (Kangwei, Beijing, China) and incubated on ice for 1 h with vortexing every 15 min and finally centrifuged at 12,000 × g for 30 min at 4 °C. Forty micrograms of protein was loaded per lane into 12% SDS-PAGE gels, separated, and transferred to 0.22 µm nitrocellulose membranes by using semi-dry blotting transfer system. Western blot was performed according to previous study [36]. After washing, bound antibodies were detected with ECL (Thermo, Waltham, MA, USA) by Bio-Imaging System (DNR Bio-Imaging Systems Ltd., Jerusalem, Israel).

### 4.5. Generation of Stable Cell Lines

PK-15 cells were co-transfected with donor vector and the helper vector expressing PB transposase. Transfected cells were cultured in fresh medium 36 h post transfection in the presence of puromycin (Merck, Darmstadt, Germany) at a concentration of 5 μg/mL. Puromycin-selective medium was then replaced every 2 days. Puromycin-resistant and GFP-positive cell clones appeared about 10 days after puromycin treatment. A single cell clone was sub-cloned into 96-well plate. As the cell reached confluence, they were split and expanded in 10-cm dishes and saved as cell stocks. The protein expression of poIRF1 in cell clone was determined by RT-qPCR and Western blot.

### 4.6. Indirect Immunofluorescence Assay (IFA)

PK-15 cells were grown to 70% confluence on coverslips and were treated by poly(I:C) stimulation or SIV infection. At 12 h post-treatment, cells were fixed with 4% paraformaldehyde and permeabilized with ice-cold acetone for 5 min. Cells were treated with a mixture of H1N1-HA antibody and poIRF1 antibody for 1 h at 37 °C in a humidified chamber followed by treatment with secondary, species-specific secondary antibody conjugated with FITC or Cy3 (Molecular Probes) for 1 h. Coverslips containing fixed samples were mounted onto slides using Vectashield containing DAPI (4′,6-diamidino-2-phenylindole) to allow visualization of cell nuclei (Vector Lab Inc., Burlingame, CA, USA). Confocal images were captured on a LSM510 META microscope (Carl Zeiss, Oberkochen, Germany).

### 4.7. Antiviral Activity Evaluation

For overexpression experiment, PK-15 cells stably expressing poIRF1 or control cells with empty vector alone were plated at a density of 4.0 × 10^5^ cells in 12 well plates. When grown to 80% confluence, cells were incubated with indicated viruses for 1 h and maintained with serum-free DMEM (containing 1 μg/mL TPCK trypsin for SIV) for 24 and 48 h before supernatants were harvested. For knockdown experiment, transfection of siRNA into PK-15 cells was performed by Hirper siRNA transfection reagents according to the manufacturer’s protocol (QIAGEN, Beijing, China). Thirty-six hours after transfection, the cells were incubated with viruses for 1 h and maintained with serum-free DMEM for 24 h before supernatants were harvested. The viral titers in supernatants were measured with 50% tissue culture infectious doses (TCID_50_) or plaque assay (plaque forming unit, PFU) in MDCK or VERO cells.

### 4.8. Luciferase Reporter Assay

To determine the impact of poIRF1 on the activation of IFN-β and ISRE promoter, the luciferase reporter plasmid IFN-β-Luc and ISRE-luc were, respectively, co-transfected in indicated cell lines with pRL-TK vector that provided the constitutive expression of Renilla luciferase (Promega) as an internal control. For knockdown experiment, PK-15 cells were transfected with si-IRF1 or si-Ctrl. After 36 h, the cells were cotransfected with IFN-β-luc or ISRE-luc and pRL-TK. At 24 h post-transfection, cells were transfected with poly(I:C) or infected with indicated viruses for another 12 h or left untreated. Then cells were lysed with 1× passive lysis buffer and the luciferase activity was detected by Dual-Luciferase Reporter Assay System (Promega) and measured with a TD-20/20 Luminometer (Turner Designs, Sunnyvale, CA, USA). Transfections were performed in triplicate and repeated at least three times in separate experiments. Error bars represent the mean SDs.
